# Leaf Multi-Element Network Reveals the Change of Species Dominance Under Nitrogen Deposition

**DOI:** 10.3389/fpls.2021.580340

**Published:** 2021-01-22

**Authors:** Jiahui Zhang, Tingting Ren, Junjie Yang, Li Xu, Mingxu Li, Yunhai Zhang, Xingguo Han, Nianpeng He

**Affiliations:** ^1^Key Laboratory of Ecosystem Network Observation and Modeling, Institute of Geographic Sciences and Natural Resources Research, Chinese Academy of Sciences, Beijing, China; ^2^College of Resources and Environment, University of Chinese Academy of Sciences, Beijing, China; ^3^State Key Laboratory of Vegetation and Environmental Change, Institute of Botany, Chinese Academy of Sciences, Beijing, China; ^4^Institute of Grassland Science, Northeast Normal University and Key Laboratory of Vegetation Ecology, Ministry of Education, Changchun, China

**Keywords:** co-vary, nitrogen addition, plasticity, species dominance, steppe, climate change

## Abstract

Elements are important functional traits reflecting plant response to climate change. Multiple elements work jointly in plant physiology. Although a large number of studies have focused on the variation and allocation of multiple elements in plants, it remains unclear how these elements co-vary to adapt to environmental change. We proposed a novel concept of the multi-element network including the mutual effects between element concentrations to more effectively explore the alterations in response to long-term nitrogen (N) deposition. Leaf multi-element networks were constructed with 18 elements (i.e., six macronutrients, six micronutrients, and six trace elements) in this study. Multi-element networks were species-specific, being effectively discriminated irrespective of N deposition level. Different sensitive elements and interactions to N addition were found in different species, mainly concentrating on N, Ca, Mg, Mn, Li, Sr, Ba, and their related stoichiometry. Interestingly, high plasticity of multi-element network increased or maintained relative aboveground biomass (species dominance) in community under simulated N deposition, which developed the multi-element network hypothesis. In summary, multi-element networks provide a novel approach for exploring the adaptation strategies of plants and to better predict the change of species dominance under altering nutrient availability or environmental stress associated with future global climate change.

## Introduction

Combined action of multiple elements supports plant biochemical reactions and adaptation to environment (Baxter, 2015; Kaspari and Powers, 2016). Macronutrients, e.g., N, P, K, Ca, Mg, and S, are necessary components for plant structure, regulation, and metabolism. Micronutrients, e.g., Fe, Mn, Zn, Cu, Ni, and Na, are essentially involved in electrochemical reactions and osmotic pressure regulation (Marschner, 2011). Trace elements, e.g., Al, Li, Ti, Co, Sr, and Ba, are toxic and negatively affect plant physiological processes, such as inhibiting growth, damaging structure, and declining biochemical activities (Cheng, 2003). Species have a relatively stable elemental composition and interrelationships in an organ under a specific environmental condition through long-term adaptation without disturbance (Sterner and Elser, 2002). Changes in elements can, therefore, reflect the allocation and adaptation strategies of plants (Zhang et al., 2018a; Zhao et al., 2020). Commonly, early studies about the function of mineral elements in plant growth and development did not consider mutual effects among elements (Chapin, 1980; An et al., 2005). This changed when Sterner and Elser (2002) proposed ecological stoichiometry, which emphasizes the strict ratios of multiple elements from the perspective of nutrient limitation (Elser et al., 2000, 2007). Recently, researchers have proposed the ionome as the elemental composition of a tissue or organism to investigate the characteristics of multiple elements one by one (Salt et al., 2008; Penuelas et al., 2019; Pillon et al., 2019). However, integral variations, which reflect the complex elemental interactions, are yet to be adequately demonstrated.

**FIGURE 1:**
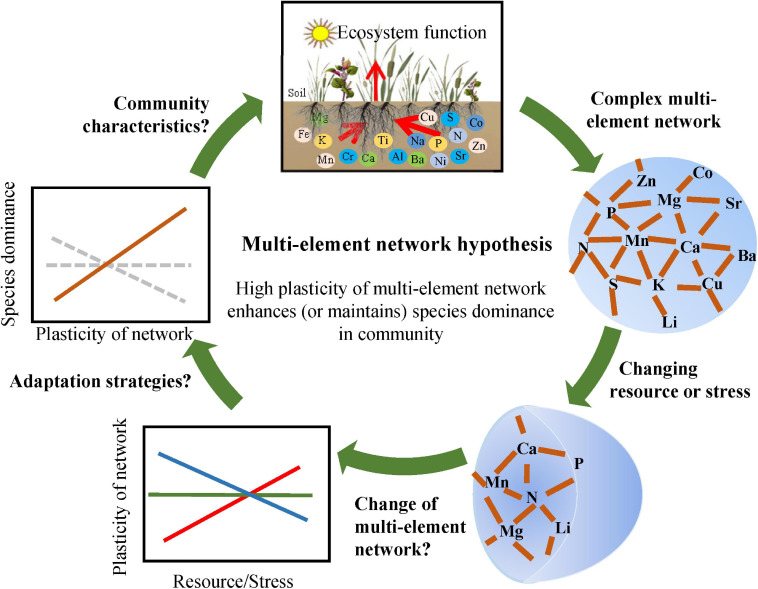
Theoretical framework for the multi-element network. Complex networks are comprised of interdependent multiple elements. Multi-element networks were supposed to be species-specific and relatively stable for a species in a particular environment without disturbance. When resource availability or environmental stress changes, multi-element networks may alter accordingly, and the plasticity of multi-element networks may be an effective strategy for adapting to the change. High plasticity enhances or maintains species dominnace, such as relative biomass (the multi-element network hypothesis).

Mineral elements in plants are responsible for different dimensional function, including water use (K and Na) (Bartels and Sunkar, 2005; Sardans and Penuelas, 2015) and photosynthesis (N, Mg, and Mn) (Tian et al., 2016). Characterizing multidimensional element structure (different principal component axes in previous studies) and identifying its ecological significance are major aims in recent research (Watanabe et al., 2007; Sardans et al., 2015). Complex networks provide a great idea to describe multidimensional systems. Some ecologists have applied complex networks to analyze the structure and function of microbial communities (Freilich et al., 2010; Ma et al., 2016) and plant traits (Flores-Moreno et al., 2019; Kleyer et al., 2019). Here, we propose a multi-element network consisting of different elements (nodes) and their interactions (links) to describe complex elemental structures (Figure 1). Multi-element networks are assumed to be species-specific owing to significant interspecific differences in element concentrations (Zhang et al., 2012; Sardans et al., 2015) and their correlations (Hao et al., 2015). Besides, relatively stable nodes and links can determine the relative stability of multi-element network for a species in a specific environment without disturbance. These properties may indicate that the change of multi-element networks comprehensively reflects the response and adaptation of plants to changing environments (Figure 1).

With enhanced N input and availability by N deposition (Galloway et al., 2008; Midgley and Phillips, 2016), N concentration in plants will increase within certain limits (Karimi and Folt, 2006), which may directly influence initial multi-element networks. Given the strong correlations between mineral nutrients, for example, P-rich RNA regulates the synthesis of N-rich protein (Sterner and Elser, 2002), and K influences the synthesis of P-rich nucleic acid (Marschner, 2011); N, P, S, Mg, Fe, and Mn are all involved in photosynthesis; Ca, Zn, and Na regulate N-involved metabolism as signals (Marschner, 2011), these nodes and links of multi-element networks may also change with increasing N availability. Trace elements are generally considered unrelated to N directly because they enter plants through osmosis rather than active uptake. However, continuously increasing N deposition will lead to soil acidification, which significantly increases available heavy metal and further changes the multi-element network (Zeng et al., 2011; Lu et al., 2014; Figure 1). Different species have different degrees of resistance to interference (Schlichting, 1986); therefore, the plasticity of multi-element network should be different among different species. Generally, the high plasticity reflects the wide range of environment in which species can survive. It is assumed that high plasticity supports the rapid response to changing environments, which makes the species more competitive. Therefore, high plasticity of multi-element network is supposed to reflect the high level of species dominance in a community, such as high relative biomass (the multi-element network hypothesis, Figure 1). Furthermore, the change of species dominance under climate change could be predicted with multi-element networks to some extent.

Leaves are the most active organ in plants. Here, data of leaf multiple elements from a long-term N deposition simulation experiment, based in a temperate steppe, was used to develop the multi-element network hypothesis. Specifically, the main objectives of this study were to answer the following questions: (1) What are the characteristics of multi-element networks? (2) How do multi-element networks respond to N deposition? (3) How does the plasticity of multi-element networks regulate species dominance in a community?

## Materials and Methods

### Plant Material and Experimental Setup

The N deposition simulation experimental site was on a temperate steppe in Inner Mongolia, China (116°40′E, 43°32′N). Mean annual temperature from 1985 to 2014 was 1.0°C with the lowest (–21.1°C) in January and the highest (19.8°C) in July. Mean annual precipitation was 350.5 mm with >70% falling during the growing season from May to August. The soil was classified as Haplic Calcisols by the Food and Agriculture Organization of the United Nations (FAO, 1985). Two C3 grasses, *Leymus chinensis* and *Stipa grandis*, accounted for >60% of aboveground biomass in the community, and were considered as dominant species (Zhang et al., 2015).

The N-addition experiment was carried out with a completely randomized block design (Zhang et al., 2014). There were nine N-addition rates tested [0 (control), 1, 2, 3, 5, 10, 15, 20, and 50 g N m^–2^ year^–1^, in the form of NH_4_NO_3_; Supplementary Table S1 and Supplementary Figure S1]. Five blocks were set as replications. Each experimental plot in blocks was 8 × 8 m^2^. All plots were separated by 1 m walkways and blocks were separated by 2 m walkways. N was added once a month on the first day of each month beginning in September 2008. It should be noted that no legumes grow in this experimental site and no fertilizer was added prior to this experiment.

Aboveground biomass was sampled by species each year from 2014 to 2018 in mid-August using a 0.5 m × 2 m quadrat (Zhang et al., 2016, 2019). We collected leaf samples of five representative species from plots to measure the element concentrations in 2018, including two dominant species (Poaceae: *L. chinensis* and *S. grandis*), two associated species (subdominant in community and associated with dominant species; Poaceae: *Agropyron cristatum* and *Achnatherum sibiricum*), and one occasional species (rare in community; Chenopodiaceae: *Chenopodium glaucum*) (Zhang et al., 2015). Soil samples were collected using a soil sampler in the 0–10 cm layers in each plot.

### Multiple Element Concentration and Soil Characteristics

All leaf samples were cleaned and oven-dried at 65°C to a constant weight and then were ground to a fine powder using a ball mill (MM400 Ball Mill, Retsch, Haan, Germany) and an agate mortar grinder (RM200, Retsch). An elemental analyzer (Vario Max CN Element Analyser, Elementar, Hanau, Germany) was used to measure N concentration. A microwave digestion system (Mars Xpress Microwave Digestion system, CEM, Matthews, NC, United States) and an inductively coupled plasma optical emission spectrometer (ICP-OES, Optima 5300 DV, Perkin Elmer, Waltham, MA, United States) were used to determine the concentration of P, K, Ca, Mg, S, Fe, Mn, Zn, Cu, Li, Na, Al, Ti, Co, Ni, Sr, and Ba (Zhang et al., 2018b; Zhao et al., 2020). Citrus leaf (GBW-10020) as a standard substance was measured to adjust random errors during digestion and measurement. In total, we measured the concentration of 18 elements for each plant species. Soil samples were air-dried and sieved using a 2 mm mesh. An Ultrameter-2 pH meter (Myron L. Company, Carlsbad, CA, United States) was used to measure soil pH.

### Statistical Analyses

Multi-element networks were constructed with different elements (nodes, elemental concentrations) and their interactions (links, elemental stoichiometry) to reflect complex multidimensional elemental structure (Figure 1). Variance analyses were used to test the effect of N addition on the concentration of each element in leaves considering the block as a random factor. General linear regressions were conducted to explore the change of elemental stoichiometry along N-addition gradient. Invariant stoichiometry indicates stable interactions in multi-element networks. To investigate whether multi-element networks are characteristic of particular species in specific environments, discriminant analyses (“MASS” package in R) were performed for all five species on 18 element concentrations in leaves for nine N-addition rates. Complete separation implies uniqueness of multi-element networks for different species, while overlap implies some similarity in multi-element networks among different species. Major discriminant elements were those represented by significantly different nodes among different multi-element networks.

Here, we developed a simple matrix-to-matrix method using interval analysis (Collins et al., 2000) to explore the response of multi-element network to nine N-addition rates. The independent variable, N-addition interval matrix, was calculated based on the differences (D) between two paired N-addition rates.

(1) $$D=x_{j}-x_{k}$$

where *x*_*j*_ is the *j*^*th*^ N-addition rate (g N m^–2^ year^–1^), and *x*_*k*_ is the *k*^*th*^ N-addition rate (g N m^–2^ year^–1^). There were 36 differences in this study.

The change in the multi-element network (dependent variable) was represented by the Euclidean distance (ED) of multiple elements as follows:

(2) $$E\,D=\sqrt{\sum_{\text{i}=1}^{T}{\left(x_{i\,j}-x_{i\,k}\right)}^{2}}$$

where *x*_*ij*_ is the relative concentration (concentration of each nutrient relative to all nutrients) of *i*^*th*^ element at the *j*^*th*^ N-addition rate, and *x*_*ik*_ is the relative concentration of *i*^*th*^ element at the *k*^*th*^ N-addition rate, and *T* is the total number of elements (18 in this study). Relative concentrations were used because multi-element networks are weighted networks. Selective absorption of plants means that the element relative concentration can represent the contribution of each element in plants and become an important weight factor (Marschner, 2011).

Furthermore, to determine the nodes that are sensitive to N deposition, N-addition interval analyses were carried out for single elements. The ED was simplified to the difference in element concentrations between different N-addition rates. Meanwhile, we normalized the element concentrations among treatments using the z-score method to strengthen the comparability of the same element under different N-addition rates as follows:

(3) $$x^{\prime }=\frac{x-\mu }{\delta }$$

where *x*′ is the standardized element concentration, *x* is the measured element concentration, μ is the mean value, and δ is the standard deviation.

In addition, we analyzed the variation of multi-element network among species along N-addition gradient as follows also using ED (Anderson et al., 2011):

(4) $$\sigma^{2}=\frac{1}{M-1}\,\left(\sum_{i,j=1}^{M}\frac{E\,D_{i\,j}^{2}}{M}\right)$$

where *σ*^2^ is the mean dissimilarity among multi-element networks of different species (interspecies variation) at a specific N-addition rate, which reflects the unevenness and complexity of spatial distribution of multi-element networks. *ED*_*ij*_ is the Euclidean distance between *i*^*th*^ and *j*^*th*^ species (*i* < *j*). *M* is the number of species at the N-addition rate. *C. glaucum* was not considered to ensure the comparability.

General linear regressions were applied among ED matrix and N-addition rate interval matrix. Positive regression lines indicate directional changes in the multi-element network or single element with N addition, non-significant regression lines indicate stochastic changes, and negative regression lines indicate that plants adopt convergent elemental strategies in N-rich environments (Figure 1). Furthermore, the slope and regression coefficient (*r*^2^) represent the direction and rate of change and the degree of noise, respectively. A steeper slope and a higher *r*^2^ indicate strong directional change and less noise (Collins et al., 2000). The slope, therefore, can be used to represent the plasticity of the multi-element network.

The change in species dominance, regarding relative aboveground biomass, reflects changes in species status or competitivity in a community. General linear regressions were used to explore the relationship between the plasticity of multi-element network and the change in relative biomass. Significant relationships indicate the regulatory effect of the multi-element network on species dominance. Otherwise, the multi-element network plays only a small role in species competitivity in community (Figure 1). The averages of relative aboveground biomass in 2014–2018 were used here to exclude other environmental disturbances as much as possible.

All analyses were performed in R software (version 3.5.1) Significance was set at *p* < 0.05.

## Results

### Characteristics of Multi-Element Networks

**FIGURE 2:**
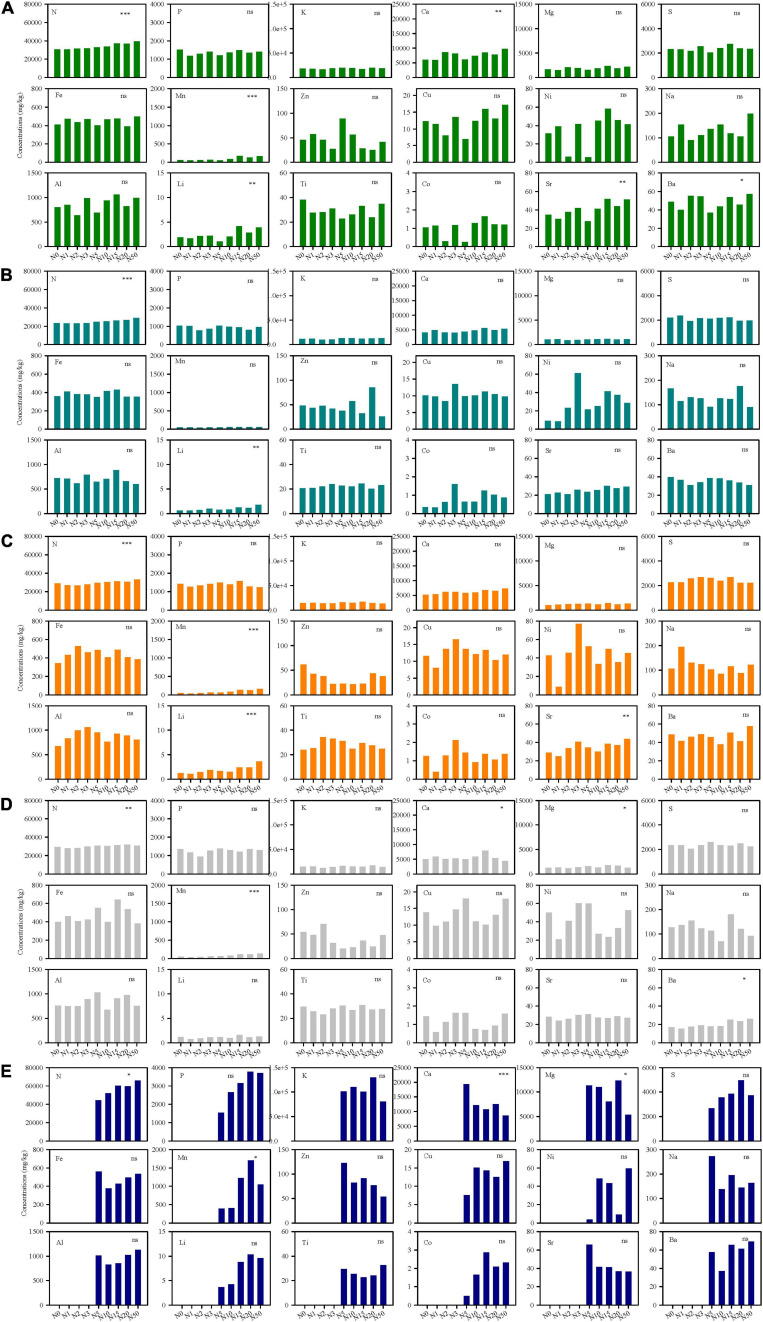
Element concentrations of five species (**A**, *Leymus chinensis*; **B**, *Stipa grandis*; **C**, *Agropyron cristatum*; **D**, *Achnatherum sibiricum*; **E**, *Chenopodium glaucum*) under different N-addition rates. Asterisks mean significant effects of N addition on the element using variance analyses. N0, N1, N2, N3, N5, N10, N15, N20, N50 mean N-addition rates at 0 (control), 1, 2, 3, 5, 10, 15, 20, and 50 g N m^–2^ year^–1^, respectively. ^∗∗∗^*p* < 0.001; ^∗∗^*p* < 0.01; ^∗^*p* < 0.05; ns, not significant.

There were significant differences of multi-element concentration and ecological stoichiometry in different species along N-addition gradient, which showed species-specific multi-element networks under different N-addition rates. The effects of N addition were significant on N, Ca, Mn, Li, Sr, and Ba in *L. chinensis*, on N and Li in *S. grandis*, on N, Mn, Li, and Sr in *A. cristatum*, on N, Ca, Mg, Mn, and Ba in *A. sibiricum*, and on N, Ca, Mg, and Mn in *C. glaucum* (Figure 2 and Supplementary Table S2). N addition rarely had a direct effect on N-related elemental stoichiometry, but mainly affected some Ca-, S-, Mn-, Li-, Sr-, and Ba-related elemental interactions (Supplementary Figures S2–S6). Moreover, the changes in stoichiometry were different among different species.

A specific species under different N-addition rates were clearly discriminated by their multi-element networks (Table 1). N was the major discriminant element for *L. chinensis*, *S. grandis*, and *C. glaucum*, while Mn was the critical one for *A. cristatum* and *A. sibiricum*. Furthermore, multi-element networks distinguished different species under a specific N-addition rate (Supplementary Figures S7, S8). Discriminant elements were distinct under each level of N addition. As N-addition rate increased, the relative positions of species in the discriminant analyses changed, indicating alterations in multi-element networks. Intersecting lines of mean centroid of each species showed the change clearly (Supplementary Figure S9). Species overlapped in the low-N-addition plots, but they were evidently distinct in the high-N-addition treatments which demonstrated the larger interspecies variation (Supplementary Figure S8).

**TABLE 1:** Discriminant analyses for the multi-element composition of species under different N-addition rates.

	Dominant species		Associated species		Occasional species	
	*Leymus*	*Stipa*	*Agropyron*	*Achnatherum*	*Chenopodium glaucum*	
	*chinensis*	*grandis*	*cristatum*	*sibiricum*		
	Score^†^	Score	Score	Score	Score 1	Score 2
N	**1.000**	**1.000**	0.199	0.164	**1.000**	−**0.012**
P	0.147	0.175	0.341	0.431	0.732	0.047
K	0.188	0.209	0.464	0.055	–0.115	–0.059
Ca	0.285	0.229	0.319	0.003	–0.495	0.005
Mg	0.351	0.219	0.555	0.362	–0.419	0.007
S	0.186	0.233	0.425	0.202	0.392	0.017
Fe	0.197	0.357	0.280	0.323	–0.373	–0.027
Mn	0.228	–0.002	**1.000**	**1.000**	0.043	0.783
Zn	0.219	–0.125	–0.108	–0.476	–0.459	–0.268
Cu	–0.317	0.064	0.264	0.373	0.701	0.076
Ni	–0.357	–0.007	0.176	0.185	0.541	0.158
Na	0.169	–0.001	0.055	0.084	–0.645	–0.456
Al	0.022	0.287	0.237	0.075	0.192	0.031
Li	0.103	–0.037	0.449	0.496	0.754	0.657
Ti	–0.067	0.171	0.271	0.179	0.237	0.243
Co	–0.350	0.021	0.163	0.180	0.652	0.224
Sr	–0.175	0.098	0.398	0.400	0.155	0.150
Ba	–0.036	0.386	0.358	0.697	–0.086	0.496
Explanation (%)	100	100	100	100	62.6	37.4

**^†^Larger score values indicated stronger discriminant effects. The bold values showed the major discriminant elements for each species.**

### Changes in Multi-Element Network With N-Addition Levels

**FIGURE 3:**
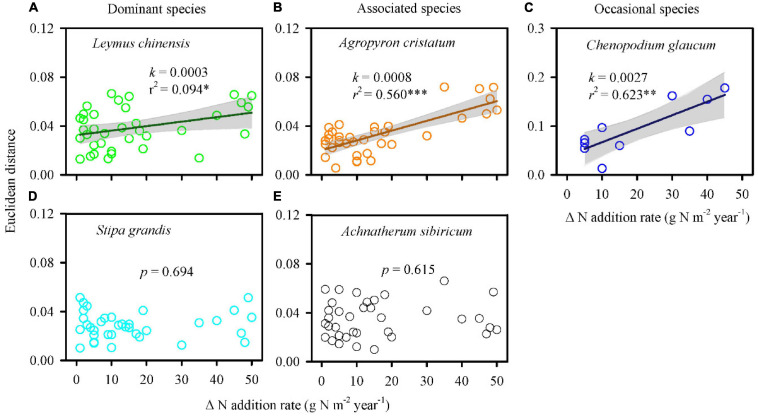
Nitrogen addition influenced the multi-element networks of different species. Euclidean distance was calculated based on 18 elements of every two paired N-addition rates. A larger Euclidean distance represented a larger change in the multi-element network. Positive regressions **(A–C)** indicated directional change. Non-significant regressions **(D,E)** represented stochastic change. A steep slope (i.e., high plasticity) and a high *r*^2^ indicated strongly directional change and less noise. *k*, slope of regression line; *r*^2^, coefficient of determination; *p*, significant level; ^∗∗∗^*p* < 0.001; ^∗∗^*p* < 0.01; ^∗^*p* < 0.05.

The multi-element networks of *L. chinensis*, *A. cristatum*, and *C. glaucum* changed directionally with increasing N-addition rate, identified by the positive regression between EDs and N-addition rate intervals (Figure 3). Specifically, low slope (0.0003) and *r*^2^ (0.094) for the regression line of *L. chinensis* indicated that directional change occurred, however, the change was slow, and stochastic variation was high. The regression lines of *A. cristatum* and *C. glaucum* exhibited high slope (0.0008 and 0.0027, respectively) and *r*^2^ (0.560 and 0.623, respectively), representing stronger signals of directional change and less noise. Conversely, the multi-element networks of *S. grandis* and *A. sibiricum* exhibited stochastic changes.

For each element node in multi-element networks, N, Ca, Mg, Mn, Cu, Na, Li, Sr, and Ba exhibited directional change in different species in the N-addition interval analyses (Table 2), in which Ca, Mg, Mn, and Li were significantly correlated with N (Supplementary Figure S10).

**TABLE 2:** N-addition interval analyses of different elements for different species.

		Dominant species				Associated species				Occasional species	
		*Leymus chinensis*		*Stipa grandis*		*Agropyron*		*Achnatherum*		*Chenopodium*	
						*cristatum*		*sibiricum*		*glaucum*	
		Slope	*r*^2†^	Slope	*r*^2^	Slope	*r*^2^	Slope	*r*^2^	Slope	*r*^2^
Macronutrient	N^‡^	0.041	0.617***	0.047	0.810***	0.034	0.489***		–		–
	Ca	0.022	0.174**		–	0.033	0.461***		–		–
	Mg		–		–		–		–	0.031	0.340*
Micronutrient	Mn	0.033	0.344***		–	0.040	0.603**	0.036	0.500***		–
	Cu	0.017	0.019*		–		–		–		–
	Na	0.032	0.352***		–		–		–		–
Trace element	Li	0.025	0.216**	0.051	0.823***	0.050	0.828**		–		–
	Sr	0.017	0.105*	0.018	0.127*	0.018	0.118**		–		–
	Ba		–		–	0.029	0.316***	0.034	0.395***		–

The independent variable was derived from the N-addition interval matrix, and the dependent variable from the Euclidean distance matrix for each element. ^†^r^2^, coefficient of determination; ***p < 0.001; **p < 0.01; *p < 0.05; –*p > 0.05. ^‡^Other macronutrients, e.g., P, K, S, micronutrients, e.g., Fe, Zn, and Ni, and trace elements, e.g., Al, Ti, Co, were not shown because of non-significant regressions for the five species.*

**FIGURE 4:**
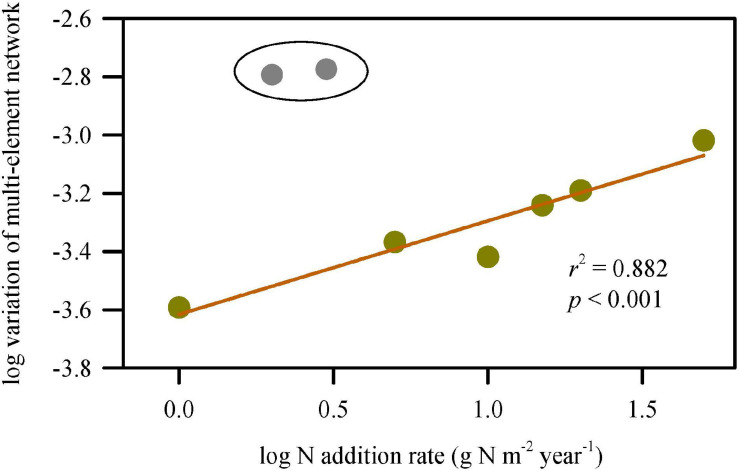
Change in variation of multi-element networks along N-addition gradient. The variations of multi-element networks were represented by *σ*^2^ which is calculated using the Euclidean distances of multiple elements between species. The variations were extremely large at the N-addition rate of 2 and 3 g N m^–2^ year^–1^. Without considering the two scenario of N addition, the variation of multi-element network significantly increased with N-addition rate.

The interspecific variation of multi-element network was extremely large at 2 and 3 g N addition m^–2^ year^–1^. Without considering the two treatments, the unevenness of multi-element networks (interspecies variation) increased significantly with increasing N-addition rate (Figure 4), which echoed the results of discriminant analyses.

### Linking the Plasticity of Multi-Element Network and the Change in Species Dominance

**FIGURE 5:**
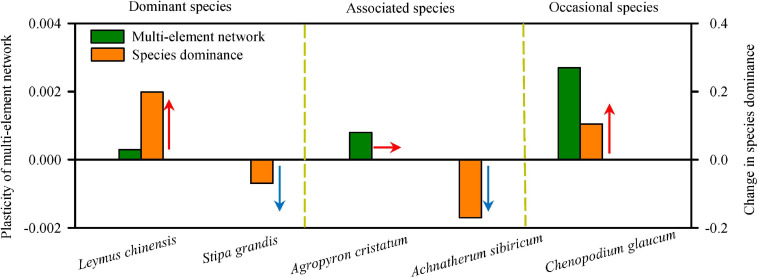
The plasticity of multi-element networks determined changes in species dominance. The plasticity of multi-element network was represented by the slope of the change of multi-element network along N-addition gradient, and the change in species dominance was represented by the slope of the relative aboveground biomass with increasing N-addition rate. High plasticity of multi-element network enhanced or maintained the species dominance in community under N addition, while low plasticity of multi-element network decreased the species dominance. Arrows showed the direction of change in species dominance.

Interestingly, the changes in the multi-element network were relatively consistent with changes in species relative aboveground biomass (species dominance in community; Figures 3, 5 and Supplementary Figure S11), which supported the multi-element network hypothesis. Those species with higher plasticity of multi-element network (e.g., *L. chinensis*, *A. cristatum*, and *C. glaucum*) showed increased or constant dominance in communities under N addition, while those with lower plasticity of multi-element network (e.g., *S. grandis* and *A. sibiricum*) showed decreased dominance.

## Discussion

### Multi-Element Networks Are Species-Specific and Discernible

Different element nodes and stoichiometric links determined discernible multi-element networks (Figure 2). Although mineral nutrients interact with each other in plant physiology (Marschner, 2011), the increasing N availability resulted from N addition only influenced several nutrient concentrations, such as N, Ca, Mg, and Mn. It indicated that Ca-regulated metabolism and Mg- and Mn-related photosynthesis were more sensitive to N addition. At the same time, soil acidification caused by N addition mainly affected the concentrations of Li, Sr, and Ba by increasing their bio-availability (Zeng et al., 2011). Above elements, therefore, were considered to be critical nodes driving the differences of multi-element networks among species under different N-addition rates. Moreover, the effect of N addition on links concentrated on changing-node-related stoichiometry rather than direct N-related stoichiometry. This result provided evidence for plant active absorption for nutrients to keep stable mutual effects. Particularly, the change of Mn-related elemental stoichiometry was generalizable, which emphasized the important role of photosynthesis in plants.

The multi-element network of a specific species was distinguishable at different N-addition levels (Table 1). The direct influence of N addition on the multi-element network resulted in N becoming discriminant elements for dominant species and occasional species in this study. Manganese was another key discriminant element, especially for associated species. These results indicated that the same node in the multi-element networks of different species may show different sensitivity to N addition. Generally, the most informative elements in the discriminant analysis were those for which considerable variation existed among species (White et al., 2012). Multi-element networks were species-specific also evidenced by the clear discrimination of different species in the same environment. As N-addition rate increased, the replications for each species increased in similarity evidenced by the gradually shrinking convex hulls in discriminant analyses (Supplementary Figure S7), and different species were easier to distinguish with multi-element networks, which echoed the increasing interspecies variation (*σ*^2^) at high N-addition rate (Figure 4) and may explain elemental niche difference to some extent (Pillon et al., 2019). Therefore, altering multi-element networks could be an adaptation strategy for plants to N deposition. In other words, environmental stress can modify the multi-element network of each species differently.

### Plasticity of Multi-Element Network Regulates Plant Response to Resource Availability

The interval analyses clearly demonstrated the changes in multi-element network along the N-addition gradient. N, Ca, Mg, Mn, Cu, Na, Li, Sr, and Ba were considered to be sensitive nodes to N addition because of the directional change (Table 2). They are all involved in important physiological processes in plants. Ca and Na as signals regulate N-involved metabolism; Mg, Mn, and Cu were essential components of chloroplast, where N-involved photosynthesis takes place (Marschner, 2011). Because Li, Sr, and Ba were not targeted by plant root and rhizosphere processes, they were considered to be sensitive to soil acidification rather than N availability. Some studies have demonstrated that random attacks on the nodes of a network do not affect the information transfer in the network, that is, the overall structure of the network here (robustness of the network) (Barabasi and Albert, 1999; Albert and Barabasi, 2002). However, targeted attacks on highly connected or sensitive nodes may lead to the disintegration of the network (Albert et al., 2000). Therefore, the physiological processes involved in sensitive nodes should be pay more attention. The corresponding change of sensitive nutrient nodes with increasing N availability supports plants to complete essential vital movement to adapt to changing environments. Therefore, a plausible explanation for the almost complete lack of *C. glaucum* in N-poor environments is based on the difficulties in establishing its multi-element network under these conditions. It is also enlightening for discussing the loss of species diversity from the perspective of multi-element network.

Increasing variation of multi-element networks with increasing N-addition rate demonstrated that different plant multi-element networks showed distinctive sensitivity to a specific N-addition rate, which indicated different plasticity of multi-element networks among species along the N-addition gradient. Furthermore, it showed that a community might respond to climate change through continuous divergence of element niches (increasing gap in demand for the same element) of different species to reduce competition. In other words, different species co-existed in a community by element niche complementarity. It should be noted that the extremely large variation of multi-element networks appeared at 2 and 3 g N addition m^–2^ year^–1^, which is close to natural N deposition (3.2 g N m^–2^ year^–1^) (Zhu et al., 2015). Calcium was the driving factor leading to the result (Figure 2). It might indicate that Ca-involved metabolism was more active in some particular species, such as *L. chinensis*, under natural N deposition.

More importantly, the plasticity of multi-element networks was closely related to the change in species relative aboveground biomass (species dominance in a community). In the present study, although both *L. chinensis* and *S. grandis* were dominant, their dominance in the community showed completely opposite trends in response to increasing N availability. The multi-element network of *L. chinensis* showed a clearly directional change (high plasticity), and its relative biomass in communities increased with N addition. However, the multi-element network of *S. grandis* did not respond strongly to N addition (low plasticity), resulting in a decrease in relative biomass. Similarly, N-addition interval analyses of associated and occasional species showed that the plasticity of multi-element network regulated plant response to nutrient availability (Figure 5). All of the evidence suggested that different species in a community exhibit different levels of multi-element network plasticity with higher plasticity increasing or maintaining species dominance under N addition, but lower plasticity decreasing species dominance. This result developed the multi-element network hypothesis and provided inspiration for exploring the change in community characteristics from an elemental perspective with climate change.

### Relevance of Multi-Element Networks

The multi-element network provides a new approach to explore plant responses to changes in resource availability by including detailed information of multiple elements. Our findings answered the questions outlined in the introduction (Figure 1) and developed an efficient interval method for describing the characteristics of multi-element networks and linking them with species dominance in a community. Using multi-element networks, researchers can identify potential changes in community characteristics not only under altering nutrient availability but also various environmental stresses. With global climate change, besides the increase in atmospheric N deposition (direct effects on multi-element networks), the change in precipitation, increasing temperature, and CO_2_ concentration will also change resource availability (indirect effects) (Sardans et al., 2008; Tian et al., 2019). Taking the change in precipitation as an example, in a warm and humid environment, moderate drought increases N absorption efficiency of plants through more root exudates (Kohli et al., 2012) and mycorrhizal symbiosis (Alvarez et al., 2009), then affects multi-element networks. In arid regions, drought reduces soil available nutrient and plant net photosynthetic rate, further leads to the redistribution of nutrients in plants, such as the decrease of N content (Sardans et al., 2008). Therefore, the development of multi-element networks will be a breakthrough for predicting the changes in species dominance and community characteristics under future global climate change.

The interval analysis provides a simple mathematical method based on ED for characterizing the plasticity of multi-element networks (Collins et al., 2000; Baez et al., 2006; He et al., 2011). It is suitable for datasets with gradients that are too short for traditional correlation analyses. Particularly, it is superior for element-addition experiments in natural environments, for which, it is difficult to set many addition rates because of the huge area requirements. This method using interval matrix increases the number of scatter in analyses to summarize trends in changing environments. Then the interval analysis considers multiple elements simultaneously and assigns a weight on the basis of element concentration to express the complex multi-element network. Furthermore, the method simplifies the complex network analysis into simple ED matrix calculations, and we can extract the rate and direction of the change and the degree of noise from the slope and *r*^2^ of the regression line (Collins et al., 2000). Combined, they help to evaluate the plasticity of multi-element network, although the evaluation method using slope depend on linear responses and is statistically limited toward comparisons of species (Valladares et al., 2006). The interval analysis works for various functional traits despite being a bit flawed in describing the whole network intuitively.

## Data Availability Statement

The raw data supporting the conclusions of this article will be made available by the authors, without undue reservation, to any qualified researcher.

## Author Contributions

JZ and NH planned and designed the research. JZ, NH, TR, JY, LX, and ML performed the experiments and conducted fieldwork. JZ, NH, TR, and JY analyzed the data. JZ, NH, TR, JY, LX, ML, YZ, and XH wrote the manuscript. All authors contributed critically to the drafts and gave final approval for publication.

## Conflict of Interest

The authors declare that the research was conducted in the absence of any commercial or financial relationships that could be construed as a potential conflict of interest.
