# Supplementary material for: Leaf Multi-Element Network Reveals the Change of Species Dominance Under Nitrogen Deposition
Source: Front Plant Sci. 2021 Jan 22;12:580340. doi: 10.3389/fpls.2021.580340 (PMC7862345; doi:10.3389/fpls.2021.580340)
Supplement: Supplementary file 1 [file Data_Sheet_1.docx]

**Table S1** Design of N addition experiments in Mongolia grasslands^1–4^

| N-addition rate  (g N m^–2^ year^–1^) | 0 | 1 | 2 | 3 | 5 | 10 | 15 | 20 | 50 |
| --- | --- | --- | --- | --- | --- | --- | --- | --- | --- |
| N-addition rate  (g N m^–2^ month^–1^) | 0 | 0.08 | 0.17 | 0.25 | 0.42 | 0.83 | 1.25 | 1.67 | 4.17 |
| NH_4_NO_3_  (g month^–1^ plot^–1^)^†^ | 0 | 15.4 | 30.7 | 45.9 | 76.6 | 153.2 | 229.7 | 306.3 | 765.8 |

^†^ Each experimental plot was 8 m × 8 m.

^1^ Zhang, Y. H., J. C. Feng, F. Isbell, X. T. Lu, and X. G. Han. 2015. Productivity depends more on the rate than the frequency of N addition in a temperate grassland. Scientific Reports 5:12558.

^2^ Zhang, Y. H., J. C. Feng, M. Loreau, N. P. He, X. G. Han, and L. Jiang. 2019. Nitrogen addition does not reduce the role of spatial asynchrony in stabilising grassland communities. Ecology Letters 22:563-571.

^3^ Zhang, Y. H., M. Loreau, X. T. Lu, N. P. He, G. M. Zhang, and X. G. Han. 2016. Nitrogen enrichment weakens ecosystem stability through decreased species asynchrony and population stability in a temperate grassland. Global Change Biology 22:1445-1455.

^4^ Zhang, Y. H., X. T. Lu, F. Isbell, C. Stevens, X. Han, N. P. He, G. M. Zhang, Q. Yu, J. H. Huang, and X. G. Han. 2014. Rapid plant species loss at high rates and at low frequency of N addition in temperate steppe. Global Change Biology 20:3520-3529.

**Table S2** F-values in variance analyses demonstrating the effect of N additon on multiple elements

|  | Dominant species |  |  |  | Associated species | |  | Occasional species |
| --- | --- | --- | --- | --- | --- | --- | --- | --- |
|  | *Leymus chinensis* | *Stipa grandis* |  |  | *Agropyron cristatum* | *Achnatherum sibiricum* |  | *Chenopodium glaucum* |
|  | F-value | F-value |  |  | F-value | F-value |  | F-value |
| N | 15.924***^†^ | 6.385*** |  |  | 7.295*** | 4.148** |  | 6.817* |
| P | 0.905 | 1.071 |  |  | 0.972 | 1.360 |  | 1.926 |
| K | 0.340 | 1.736 |  |  | 0.811 | 1.683 |  | 5.209 |
| Ca | 4.576** | 1.824 |  |  | 0.860 | 2.761* |  | 69.474*** |
| Mg | 1.799 | 0.988 |  |  | 1.689 | 2.794* |  | 9.678* |
| S | 1.231 | 0.712 |  |  | 1.800 | 0.553 |  | 2.887 |
| Fe | 0.481 | 0.839 |  |  | 1.090 | 1.722 |  | 0.970 |
| Mn | 5.660*** | 0.942 |  |  | 20.303*** | 9.943*** |  | 6.153* |
| Zn | 1.295 | 0.567 |  |  | 0.784 | 0.937 |  | 0.460 |
| Cu | 1.481 | 0.456 |  |  | 1.251 | 1.170 |  | 0.462 |
| Ni | 0.640 | 1.233 |  |  | 1.825 | 0.512 |  | 0.897 |
| Na | 0.778 | 1.371 |  |  | 1.521 | 1.188 |  | 0.278 |
| Al | 0.890 | 0.766 |  |  | 0.960 | 0.918 |  | 0.686 |
| Li | 4.387** | 4.380** |  |  | 29.298*** | 0.995 |  | 4.771 |
| Ti | 1.629 | 0.329 |  |  | 1.726 | 0.583 |  | 0.142 |
| Co | 0.592 | 1.226 |  |  | 1.712 | 0.554 |  | 0.810 |
| Sr | 4.605** | 1.321 |  |  | 4.174** | 0.344 |  | 0.999 |
| Ba | 2.787* | 0.864 |  |  | 1.439 | 2.938* |  | 2.601 |

^†^ ***, *p* < 0.001; **, *p* < 0.01; *, *p* < 0.05.

**Fig. S1** Schematic diagram of the N-addition completely randomized block design.

Nine N-addition rates were tested, including 0 (control), 1, 2, 3, 5, 10, 15, 20, and 50 g N m^–2^ year^–1^, in the form of NH_4_NO_3_. Each experimental plot representing one kind of N-addition treatment was 8 × 8 m^2^. Five blocks were set as replications. All plots were separated by 1m walkways and blocks were separated by 2 m walkways.

**Fig. S2** Changes in elemental stoichiometry of *Leymus chinensis* with increasing N-addition rates.

**Fig. S3** Changes in elemental stoichiometry of *Stipa grandis* with increasing N-addition rates.

**Fig. S4** Changes in elemental stoichiometry of *Agropyron cristatum* with increasing N-addition rates.

**Fig. S5** Changes in elemental stoichiometry of *Achnatherum sibiricum* with increasing N-addition rates.

**Fig. S6** Changes in elemental stoichiometry of *Chenopodium glaucum* with increasing N-addition rates.


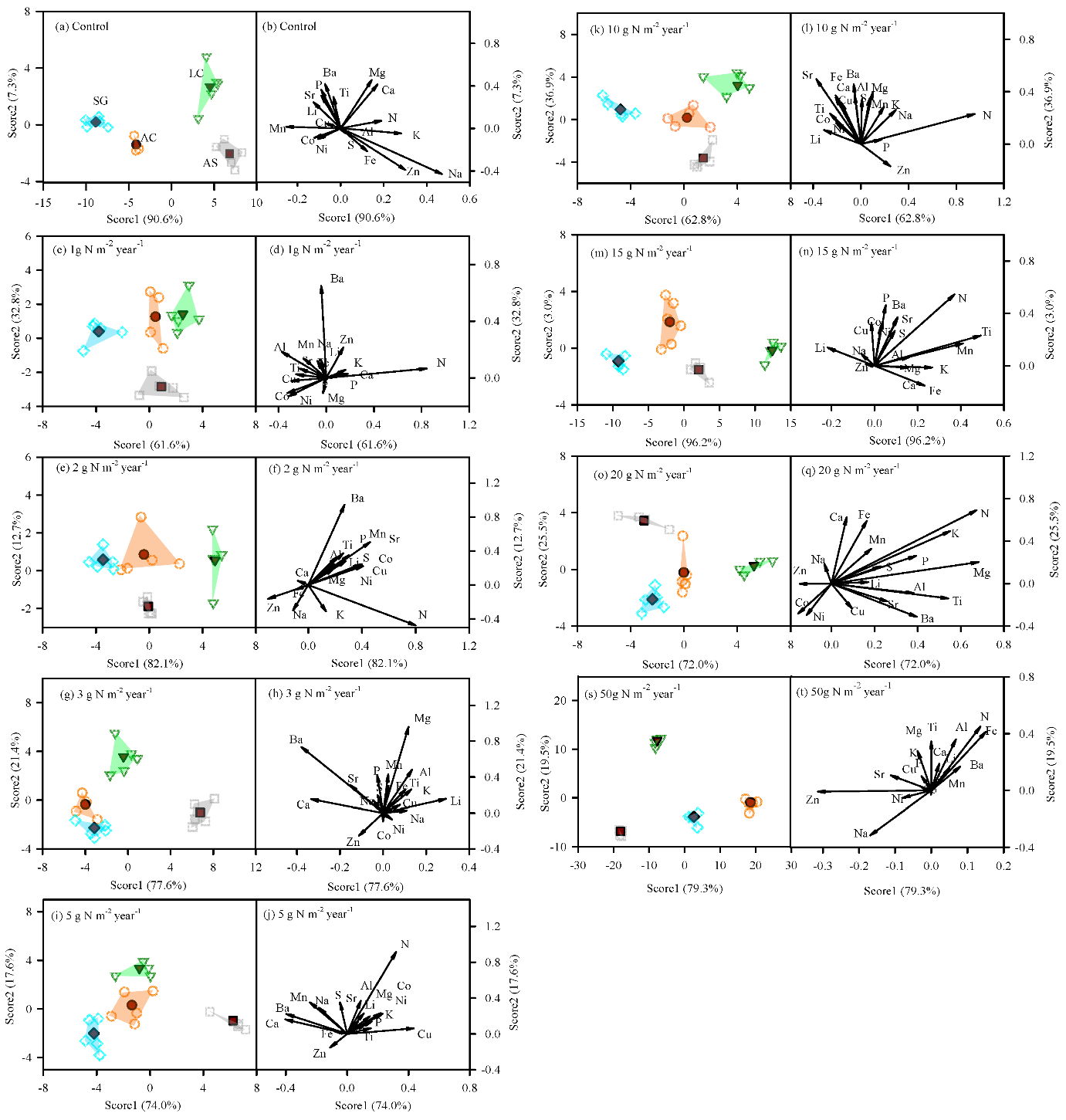


**Fig. S7** Diverse plant species can be discriminated by their multi-element networks at each N-addition rate. LC, *Leymus chinensis*; SG, *Stipa grandis*; AC, *Agropyron cristatum*; AS, *Achnatherum sibiricum*. Dark red shapes represent the mean centroid of each species. Convex hulls of each species are shown in the figure. *Chenopodium glaucum* was not included here to ensure the comparability, because few *Chenopodium glaucum* were observed in low-N-addition plots.


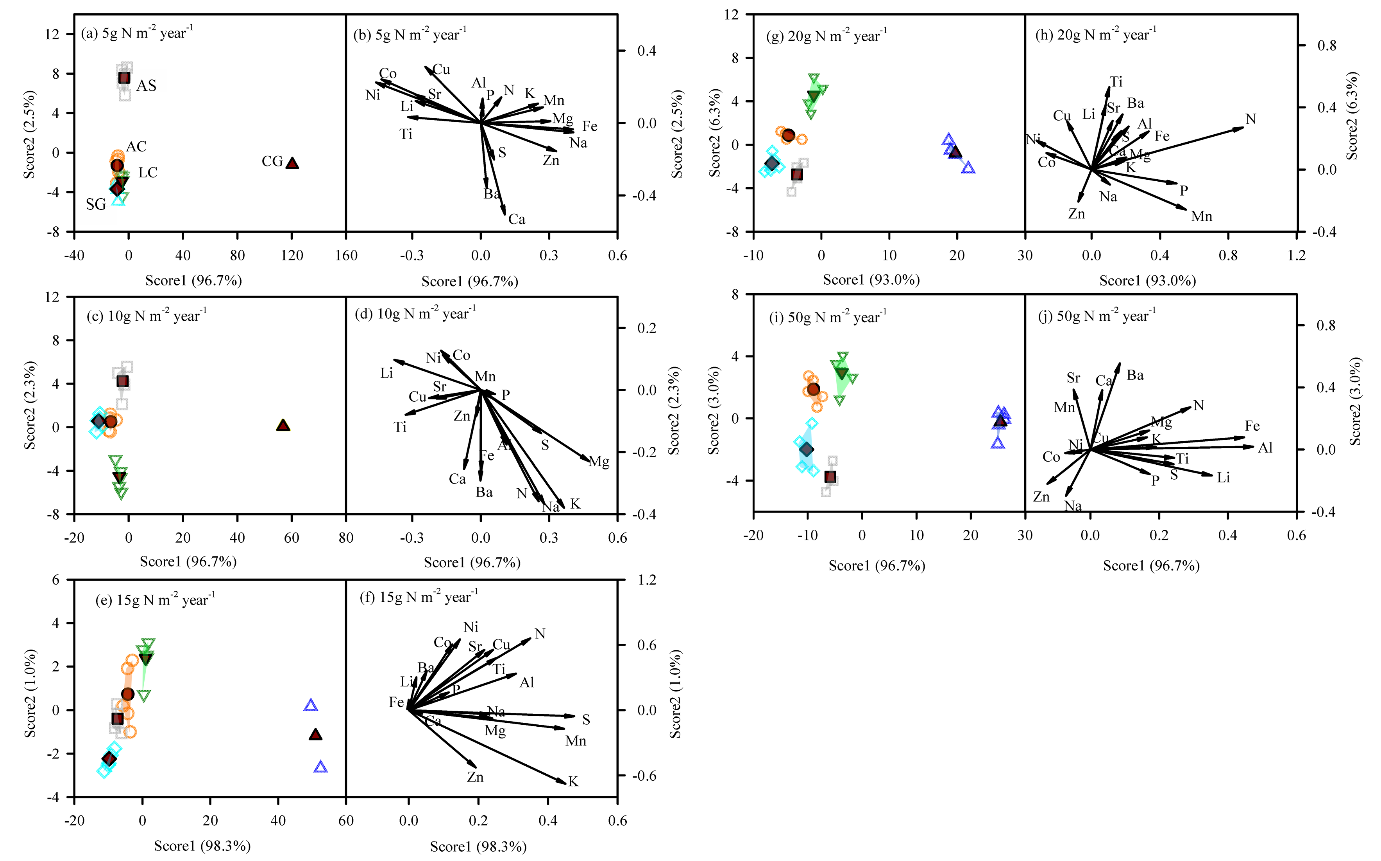


**Fig. S8** Discriminant analyses for five species using multi-element networks at high-N-addition treatments. LC, *Leymus chinensis*; SG, *Stipa grandis*; AC, *Agropyron cristatum*; AS, *Achnatherum sibiricum*; CG, *Chenopodium glaucum*. Dark red shapes represent the mean centroid of each species. Convex hulls of each species are shown.

**Fig. S9** Change of mean centroid in discriminant analyses with N-addition rate. Intersecting lines mean the change of relative positions of four species in the discriminant analyses, further, the alterations in multi-element networks with N-addition rate. *Chenopodium glaucum* was not included to ensure the comparability, because few *Chenopodium glaucum* were observed in low-N-addition plots.


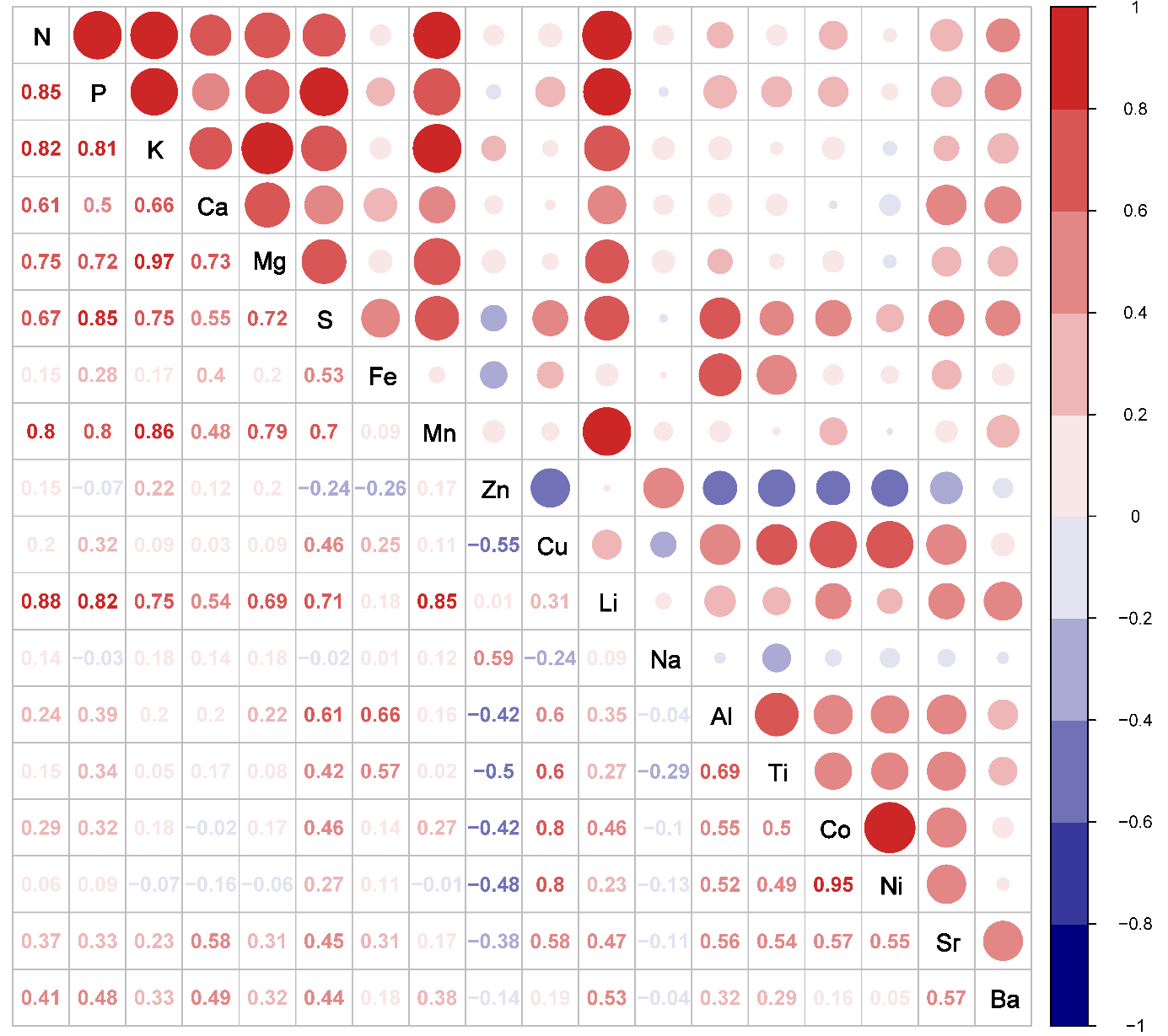


**Fig. S10** Correlation coefficients matrix among different elements

Red color means positive correlation, and blue means negative correlation. Larger and darker circles indicate stronger correlation.

**Fig. S11** Changes in relative aboveground biomass with increasing N-addition rates. The triangles indicate the relative aboveground biomass at the control level. *r*^2^, coefficient of determination; *p*, significance level.

**Fig. S12** Soil pH significantly decreased with increasing N-addition rates. *r*^2^, coefficient of determination, *p*, significance level.
